# Role of Klotho in Chronic Calcineurin Inhibitor Nephropathy

**DOI:** 10.1155/2019/1825018

**Published:** 2019-10-17

**Authors:** Kang Luo, Sun Woo Lim, Yi Quan, Sheng Cui, Yoo Jin Shin, Eun Jeong Ko, Byung Ha Chung, Chul Woo Yang

**Affiliations:** ^1^Transplant Research Center, Seoul St. Mary's Hospital, College of Medicine, The Catholic University of Korea, Seoul, Republic of Korea; ^2^Convergent Research Consortium for Immunologic Disease, Seoul St. Mary's Hospital, College of Medicine, The Catholic University of Korea, Seoul, Republic of Korea; ^3^Division of Nephrology, Department of Internal Medicine, Seoul St. Mary's Hospital, College of Medicine, The Catholic University of Korea, Seoul, Republic of Korea; ^4^Department of Nephrology, Yanbian University Hospital, Yanbian, China

## Abstract

Calcineurin inhibitors (CNIs) are the most popular immunosuppressants in organ transplantation, but nephrotoxicity is a major concern. The common mechanism underlying chronic CNI nephropathy is oxidative stress, and the process of chronic CNI nephropathy is similar to that of aging. Current studies provide evidence that antiaging Klotho protein plays an important role in protecting against oxidative stress, and its signaling is a target for preventing oxidative stress-induced aging process. In this review, we focus on the association between Klotho and oxidative stress and the protective mechanism of action of Klotho against oxidative stress in chronic CNI nephropathy. In addition, we discuss the delivery strategy for Klotho in CNI-induced nephropathy.

## 1. Overview of Klotho in Human Disease

Klotho is an aging-suppressor gene [1, 2], and it encodes a single-pass transmembrane protein. The extracellular domain of Klotho protein is cleaved on the cell surface by membrane-anchored proteases and is released into the blood [3–5], urine [6–8], and cerebrospinal fluid [4]. Secreted Klotho proteins have diverse functions, including the regulation of multiple ion channels [6, 8–10] and oxidative stress [11–13].

Klotho is involved in various pathologies, such as atherosclerosis, heart failure, hypertension, acute kidney injury, chronic kidney disease, diabetes mellitus, and even cancer [14–17]. Interestingly, Klotho is highly expressed in the kidney [1], and its expression is suppressed under sustained stress conditions in several animal models [18–22] of kidney injury and in patients with chronic renal failure [23]. Thus, the role of Klotho in kidney injury has attracted increasing attention from researchers.

## 2. Overview of Chronic CNI Nephropathy

Calcineurin inhibitors (CNIs) are the most popular immunosuppressive drugs used for solid organ transplantation, and two CNIs [cyclosporine (CsA) and tacrolimus (TAC)] are available in clinical practice [24]. CNI exerts its immunosuppressive action by inhibiting calcineurin in T-cells. This inhibition then impairs translocation of the nuclear factor of activated T-cells [25–27], which regulates IL-2 transcription and thus T-cell activation [28–30]. Despite the specific inhibition of T-cell activation, long-term treatment with CNIs causes serious adverse effects, and nephrotoxicity is a major issue in solid organ transplantation.

Utilizing a well-established animal model, we and others have demonstrated that CNI causes low-grade ischemic injury by reducing renal blood follow and activating a complex network of proinflammatory and profibrotic mediators (for example, osteopontin [31, 32] and transforming growth factor *β*1 [33, 34]), along with the renin-angiotensin system [35, 36], apoptosis [37, 38], and endothelial dysfunction [39]. The oxidative stress caused by reactive oxygen species (ROS) is regarded as a common pathway of CNI-induced nephrotoxicity. Antioxidative agents such as statin, angiotensin II blockade, or N-acetylcysteine are known to improve CNI-induced renal injury [40–43].

**Figure 1:**
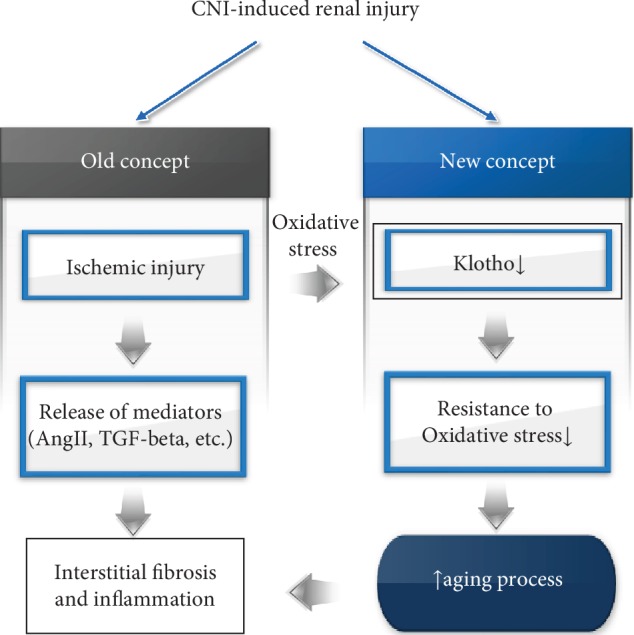
The concept of chronic CNI nephropathy as aging process. Low-grade ischemic injury by long-term CNI treatment decreases antiaging Klotho protein. Thus, renal tubular cells lost its ability to resistance to oxidative stress and subsequent cell death occurs.

Chronic CNI nephropathy causes progressive renal failure [44] which is similar to alterations that occur with aging. Indeed, telomere shortening and upregulation of senescence-associated cell cycle inhibitors were reported in CNI-treated renal tubular cells [45] and in renal transplants with graft dysfunction [46]. Thus, we proposed that oxidative stress due to low-grade ischemia accelerates the aging process in chronic CNI nephropathy and the antiaging protein Klotho may be involved in this process (Figure 1).

## 3. Klotho Expression and Oxidative Stress in Chronic CNI Nephropathy

Using animal model of chronic CNI nephropathy, we firstly reported that CNI treatment decreased Klotho mRNA and protein in the mouse kidney in a dose- and time-dependent manner [43, 47] and Klotho expression was correlated with activity of renin-angiotensin system, tubulointerstitial fibrosis, and marker of oxidative stress (urinary 8-hydroxy-2′-deoxyguanosine (8-OHdG) excretion) [48]. This finding suggests that long-term treatment of CNI decreases Klotho expression in the kidney and Klotho is a useful marker to represent chronic CNI nephropathy.

A Klotho-deficient mouse aging model is useful to define the causal relationship between oxidative stress and Klotho. Kuro-o et al. reported that Klotho deficiency is closely related to cardiovascular diseases [1] and Klotho is an important humoral factor involved in oxidative stress regulation, endothelial dysfunction, cell proliferation, and apoptosis [49–51]. Using Klotho +/− mice, we found that Klotho deficiency renders the kidney more susceptible to TAC-induced injury, which was closely associated with aggravated TAC-induced oxidative stress [47]. These findings suggest strong associations between Klotho and CNI-induced oxidative stress and provide evidence that Klotho plays an important role in protecting against CNI-induced oxidative stress.

## 4. Protective Mechanism of Action of Klotho against CNI-Induced Oxidative Stress

Klotho is involved in several intracellular signaling pathways (PKC, FGF23, cAMP, TGF-*β*, p53/p21, Wnt signaling, and PDLIM2/NF-*κ*B p65 pathway) [52, 53], and many studies have reported the interactions among these pathways [54, 55]. In this review, we focus on the antioxidative function of Klotho via the intracellular phosphatidylinositol 3-kinase (PI3K)-Akt serine-threonine kinase (AKT) signaling pathway.

The PI3K-AKT signaling pathway regulates forkhead box protein O (FoxO) through phosphorylation. The AKT-mediated phosphorylation of FoxO inhibits FoxO activity by promoting its interaction with 14-3-3 proteins and nuclear exportation and also by inducing its proteasomal degradation [56]. FoxO3a can upregulate manganese superoxide dismutase (MnSOD) expression [2, 57, 58]. Thus, FoxO3a functions as a negative regulator of mitochondrial ROS production [59] and thereby closely associates with resistance to oxidative stress. In an experimental model of TAC-induced nephropathy, we found that concomitant Klotho treatment inhibits the PI3K/AKT-mediated phosphorylation of FoxO3a and enhances FoxO3a binding to the MnSOD promoter. Thus, Klotho increases MnSOD mRNA and protein expression in mitochondria and reduces TAC-induced mitochondrial dysfunction and ROS production [60]. Taken together, Klotho protects TAC-induced oxidative stress by negatively regulating the PI3K/AKT pathway and subsequently enhances FoxO3a-mediated MnSOD expression.

## 5. Role of Klotho in CNI-Induced Cell Death

Endoplasmic reticulum (ER) stress, a common cellular stress, is a potent trigger for autophagy, which is an important protective mechanism against various cellular stresses, including nutrient deprivation, hypoxia, and growth factor deprivation [61, 62]. Thus, the balance between ER stresses and autophagy is important to maintain cell viability, and excessive ER stress or impaired autophagy may cause apoptotic cell death. Recent reports showed that Klotho plays an important role in modulating ER signaling crosstalk between autophagy and apoptosis [49–51] and Klotho treatment alleviates ER stress in unilateral ureteral obstruction or attenuates oxidant-induced alveolar epithelial cell apoptosis [63]. In addition, the association between Klotho and autophagy has been reported in various diseases, such as Alzheimer's disease, acute kidney injury, chronic obstructive pulmonary disease, and lung cancer [64–67].

**Figure 2:**
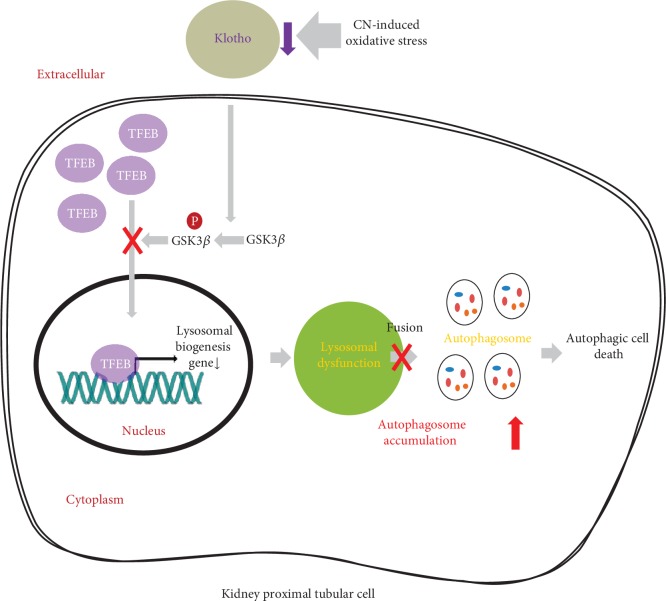
The protective mechanism of Klotho in CNI-induced autophagic cell death. Klotho induces nuclear translocation of transcription factor EB (TFEB), a master regulator for lysosomal biogenesis, through inhibition of phosphorylation of glycogen synthase kinase 3*β* (GSK3*β*). Improved lysosomal function by Klotho increases clearance of autophagosome and resulted in decrease of autophagic cell death.

CNI-induced renal injury involves induction of the ER stress response and apoptosis [68, 69]. Kidneys treated with CNI for a short time adapt well to such stress by synthesizing molecular chaperones and activating autophagy process. However, prolonged ER stress by CNI exposure may cause apoptosis by depleting molecular chaperones and overloaded autophagosome [70, 71]. We recently reported that chronic CNI nephropathy is a state of excessive accumulation of autophagosome and impaired autophagy clearance [72] and Klotho treatment reduces the burden of autophagy vacuoles by improving autophagy clearance via activation of lysosomal function in CNI-induced nephrotoxicity [73]. We summarized the mechanism of protective effect of Klotho on CNI-induced autophagy cell death in Figure 2.

## 6. Delivering Strategy for Klotho

We and other researchers studied how to preserve Klotho against oxidative stress in kidney, and we reported that angiotensin II blockade, statin, and N-acetylcysteine are effective in preserving Klotho in experimental model of chronic CNI nephropathy [40, 42, 43]. However, it is not certain whether preservation of Klotho by these drugs is casually related to the antioxidant effect.

**Figure 3:**
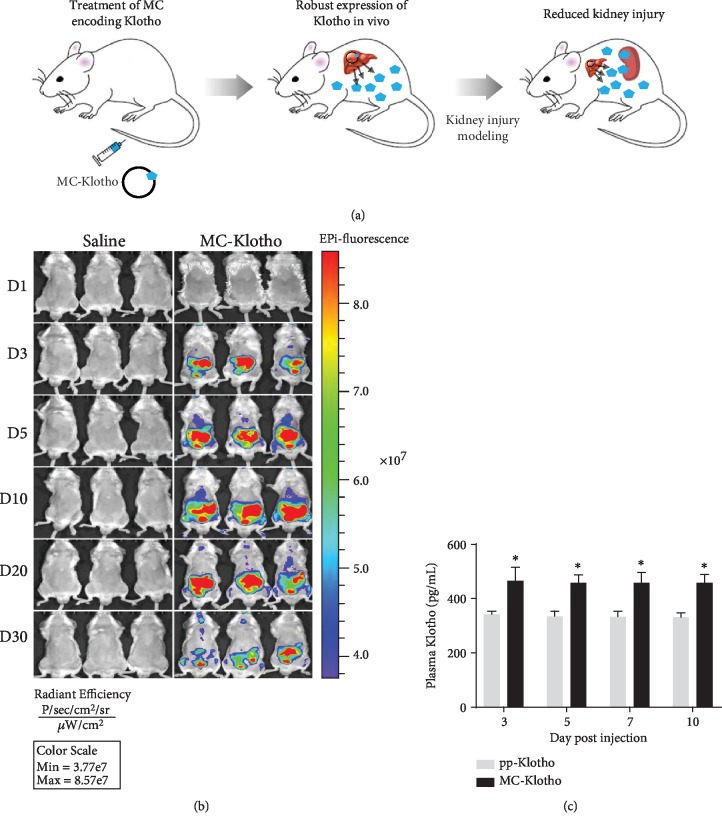
Strategy of Klotho delivery using minicircle vector system. (a) Production of in vivo Klotho using minicircle vector system. (b) Representative pictures of mice with Klotho protein derived from minicircles in vivo, each day after injection of saline or MC-Klotho using in vivo imaging system. Note that red fluorescence protein signal can be observed at day 30. (c) The plasma level of Klotho by ELISA. Saline-treated group was used as a negative control. pp: parental plasmid DNA; MC: minicircle plasmid DNA. ^#^*P* < 0.05 vs. the other group. ∗*P* < 0.05 vs. corresponding pp-Klotho. Scale bar = 100 *μ*m.

Accumulating evidence indicates that administration of exogenous Klotho is a rational strategy for the treatment of acute/chronic kidney diseases [74]. However, the half-life of recombinant Klotho is so short (7.2 h) that frequent injection (every day or every alternative day) is needed to achieve therapeutic efficacy [60, 75–77]. To overcome this limitation, we developed minicircle (MC) vector encoding Klotho protein. Using MC delivery, we can detect MC-Klotho until 30 days and MC-mediated Klotho protein until 10 days after single injection via the tail vein and at significantly higher levels than that of conventional vectors [78] (Figure 3). Thus, the MC-mediated vector encoding Klotho provides more long-term and stable Klotho expression than recombinant Klotho protein. We observed the effect of MC in an animal model of ischemia-reperfusion injury and obstructive nephropathy [78]. We expect that MC-mediated Klotho protein production may offer a new approach to Klotho delivery in clinical practice.

## 7. Conclusions

Klotho plays an important role in protecting against CNI-induced oxidative stress. Klotho and its signaling is an important target of preventing oxidative stress-induced organ injury.

## References

[1] Kuro-o M., Matsumura Y., Aizawa H. (1997). Mutation of the mouse *klotho* gene leads to a syndrome resembling ageing. *Nature*.

[2] Kurosu H., Yamamoto M., Clark J. D. (2005). Suppression of aging in mice by the hormone Klotho. *Science*.

[3] Imura A., Tsuji Y., Murata M. (2007). *α*-Klotho as a regulator of calcium homeostasis. *Science*.

[4] Imura A., Iwano A., Tohyama O. (2004). Secreted Klotho protein in sera and CSF: implication for post-translational cleavage in release of Klotho protein from cell membrane. *FEBS Letters*.

[5] Li S. A., Watanabe M., Yamada H., Nagai A., Kinuta M., Takei K. (2004). Immunohistochemical localization of Klotho protein in brain, kidney, and reproductive organs of mice. *Cell Structure and Function*.

[6] Chang Q., Hoefs S., van der Kemp A. W., Topala C. N., Bindels R. J., Hoenderop J. G. (2005). The *β*-glucuronidase klotho hydrolyzes and activates the TRPV5 channel. *Science*.

[7] Hu M. C., Shi M., Zhang J. (2011). Klotho deficiency causes vascular calcification in chronic kidney disease. *Journal of the American Society of Nephrology*.

[8] Hu M. C., Shi M., Zhang J., Quinones H., Kuro-o M., Moe O. W. (2010). Klotho deficiency is an early biomarker of renal ischemia–reperfusion injury and its replacement is protective. *Kidney International*.

[9] Cha S. K., Ortega B., Kurosu H., Rosenblatt K. P., Kuro O. M., Huang C. L. (2008). Removal of sialic acid involving Klotho causes cell-surface retention of TRPV5 channel via binding to galectin-1. *Proceedings of the National Academy of Sciences*.

[10] Cha S. K., Hu M. C., Kurosu H., Kuro-o M., Moe O., Huang C. L. (2009). Regulation of renal outer medullary potassium channel and renal K^+^ excretion by Klotho. *Molecular Pharmacology*.

[11] Kuro-o M. (2008). Klotho as a regulator of oxidative stress and senescence. *Biological Chemistry*.

[12] Mitobe M., Yoshida T., Sugiura H., Shirota S., Tsuchiya K., Nihei H. (2005). Oxidative stress decreases klotho expression in a mouse kidney cell line. *Nephron Experimental Nephrology*.

[13] Shimizu H., Bolati D., Adijiang A. (2011). Indoxyl sulfate downregulates renal expression of Klotho through production of ROS and activation of nuclear factor-*κ*B. *American Journal of Nephrology*.

[14] Donate-Correa J., Martin-Nunez E., Mora-Fernandez C., Muros-de-Fuentes M., Perez-Delgado N., Navarro-Gonzalez J. F. (2015). Klotho in cardiovascular disease: current and future perspectives. *World Journal of Biological Chemistry*.

[15] Zhou X., Wang X. (2015). Klotho: a novel biomarker for cancer. *Journal of Cancer Research and Clinical Oncology*.

[16] Jeong S. J., Song J. E., Kim S. B. (2013). Plasma klotho levels were inversely associated with subclinical carotid atherosclerosis in HIV-infected patients receiving combined antiretroviral therapy. *AIDS Research and Human Retroviruses*.

[17] Chang J. R., Sun N., Nan Y., Yu W., Qi Y. F. (2015). Research progress of Klotho. *Sheng Li Ke Xue Jin Zhan*.

[18] Aizawa H., Saito Y., Nakamura T. (1998). Downregulation of the *Klotho* gene in the kidney under sustained circulatory stress in rats. *Biochemical and Biophysical Research Communications*.

[19] Mitani H., Ishizaka N., Aizawa T. (2002). In vivo *klotho* gene transfer ameliorates angiotensin II-induced renal damage. *Hypertension*.

[20] Nagai R., Saito Y., Ohyama Y. (2000). Endothelial dysfunction in the klotho mouse and downregulation of klotho gene expression in various animal models of vascular and metabolic diseases. *Cellular and Molecular Life Sciences*.

[21] Sugiura H., Yoshida T., Tsuchiya K. (2005). Klotho reduces apoptosis in experimental ischaemic acute renal failure. *Nephrology Dialysis Transplantation*.

[22] Vonend O., Apel T., Amann K. (2004). Modulation of gene expression by moxonidine in rats with chronic renal failure. *Nephrology Dialysis Transplantation*.

[23] Koh N., Fujimori T., Nishiguchi S. (2001). Severely reduced production of klotho in human chronic renal failure kidney. *Biochemical and Biophysical Research Communications*.

[24] Andreoni K. A., Brayman K. L., Guidinger M. K., Sommers C. M., Sung R. S. (2007). Kidney and pancreas transplantation in the United States, 1996–2005. *American Journal of Transplantation*.

[25] Flanagan W. M., Corthesy B., Bram R. J., Crabtree G. R. (1991). Nuclear association of a T-cell transcription factor blocked by FK-506 and cyclosporin A. *Nature*.

[26] Jain J., McCaffrey P. G., Miner Z. (1993). The T-cell transcription factor NFATp is a substrate for calcineurin and interacts with Fos and Jun. *Nature*.

[27] Shaw K. T., Ho A. M., Raghavan A. (1995). Immunosuppressive drugs prevent a rapid dephosphorylation of transcription factor NFAT1 in stimulated immune cells. *Proceedings of the National Academy of Sciences of the United States of America*.

[28] Clipstone N. A., Crabtree G. R. (1992). Identification of calcineurin as a key signalling enzyme in T-lymphocyte activation. *Nature*.

[29] O'Keefe S. J., Tamura J., Kincaid R. L., Tocci M. J., O'Neill E. A. (1992). FK-506- and CsA-sensitive activation of the interleukin-2 promoter by calcineurin. *Nature*.

[30] Emmel E. A., Verweij C. L., Durand D. B., Higgins K. M., Lacy E., Crabtree G. R. (1989). Cyclosporin A specifically inhibits function of nuclear proteins involved in T cell activation. *Science*.

[31] Li C., Yang C. W., Park J. H. (2004). Pravastatin treatment attenuates interstitial inflammation and fibrosis in a rat model of chronic cyclosporine-induced nephropathy. *American Journal of Physiology-Renal Physiology*.

[32] Li C., Yang C. W., Kim W. Y. (2003). Reversibility of chronic cyclosporine nephropathy in rats after withdrawal of cyclosporine. *American Journal of Physiology-Renal Physiology*.

[33] Li C., Lim S. W., Choi B. S. (2005). Inhibitory effect of pravastatin on transforming growth factor *β*1-inducible gene h3 expression in a rat model of chronic cyclosporine nephropathy. *American Journal of Nephrology*.

[34] Sun B. K., Li C., Lim S. W. (2004). Expression of transforming growth factor-*β*–inducible gene-h3 in normal and cyclosporine-treated rat kidney. *Journal of Laboratory and Clinical Medicine*.

[35] Yang C. W., Ahn H. J., Kim W. Y. (2001). Influence of the renin-angiotensin system on epidermal growth factor expression in normal and cyclosporine-treated rat kidney. *Kidney International*.

[36] Sun B. K., Li C., Lim S. W. (2005). Blockade of angiotensin II with losartan attenuates transforming growth factor-*β*1 inducible gene-h3 (*β*ig-h3) expression in a model of chronic cyclosporine nephrotoxicity. *Nephron Experimental Nephrology*.

[37] Yang C. W., Faulkner G. R., Wahba I. M. (2002). Expression of apoptosis-related genes in chronic cyclosporine nephrotoxicity in mice. *American Journal of Transplantation*.

[38] Li C., Lim S. W., Sun B. K. (2004). Expression of apoptosis-related factors in chronic cyclosporine nephrotoxicity after cyclosporine withdrawal. *Acta Pharmacologica Sinica*.

[39] Yang C. W., Kim Y. S., Kim J. (1998). Oral supplementation of L-arginine prevents chronic cyclosporine nephrotoxicity in rats. *Nephron Experimental Nephrology*.

[40] Piao S. G., Kang S. H., Lim S. W. (2013). Influence of *N*-acetylcysteine on *Klotho* expression and its signaling pathway in experimental model of chronic cyclosporine nephropathy in mice. *Transplantation Journal*.

[41] Yoon H. E., Choi B. S. (2014). The renin-angiotensin system and aging in the kidney. *The Korean Journal of Internal Medicine*.

[42] Yoon H. E., Lim S. W., Piao S. G., Song J. H., Kim J., Yang C. W. (2012). Statin upregulates the expression of *klotho*, an anti-aging gene, in experimental cyclosporine nephropathy. *Nephron Experimental Nephrology*.

[43] Yoon H. E., Ghee J. Y., Piao S. (2011). Angiotensin II blockade upregulates the expression of Klotho, the anti-ageing gene, in an experimental model of chronic cyclosporine nephropathy. *Nephrology Dialysis Transplantation*.

[44] Yoon H. E., Yang C. W. (2009). Established and newly proposed mechanisms of chronic cyclosporine nephropathy. *The Korean Journal of Internal Medicine*.

[45] Jennings P., Koppelstaetter C., Aydin S. (2007). Cyclosporine A induces senescence in renal tubular epithelial cells. *American Journal of Physiology-Renal Physiology*.

[46] Melk A., Schmidt B. M., Vongwiwatana A., Rayner D. C., Halloran P. F. (2005). Increased expression of senescence-associated cell cycle inhibitor p16*^INK4a^* in deteriorating renal transplants and diseased native kidney. *American Journal of Transplantation*.

[47] Jin J., Jin L., Lim S. W., Yang C. W. (2016). Klotho deficiency aggravates tacrolimus-induced renal injury via the phosphatidylinositol 3-kinase-Akt-forkhead box protein O pathway. *American Journal of Nephrology*.

[48] Hu M. C., Kuro-o M., Moe O. W. (2013). Klotho and chronic kidney disease. *Contributions to Nephrology*.

[49] Kokkinaki M., Abu-Asab M., Gunawardena N. (2013). Klotho regulates retinal pigment epithelial functions and protects against oxidative stress. *The Journal of Neuroscience*.

[50] Kuro-o M. (2011). Klotho and the aging process. *The Korean Journal of Internal Medicine*.

[51] Rakugi H., Matsukawa N., Ishikawa K. (2007). Anti-oxidative effect of Klotho on endothelial cells through cAMP activation. *Endocrine*.

[52] Sopjani M., Rinnerthaler M., Kruja J., Dermaku-Sopjani M. (2015). Intracellular signaling of the aging suppressor protein Klotho. *Current Molecular Medicine*.

[53] Imai M., Ishikawa K., Matsukawa N. (2004). Klotho protein activates the PKC pathway in the kidney and testis and suppresses 25-hydroxyvitamin D_3_ 1*α*-hydroxylase gene expression. *Endocrine*.

[54] Freudlsperger C., Bian Y., Contag Wise S. (2013). TGF-*β* and NF-*κ*B signal pathway cross-talk is mediated through TAK1 and SMAD7 in a subset of head and neck cancers. *Oncogene*.

[55] Jin B., Wang C., Li J., Du X., Ding K., Pan J. (2017). Anthelmintic niclosamide disrupts the interplay of p65 and FOXM1/*β*-catenin and eradicates leukemia stem cells in chronic myelogenous leukemia. *Clinical Cancer Research*.

[56] Tzivion G., Dobson M., Ramakrishnan G. (2011). FoxO transcription factors; regulation by AKT and 14-3-3 proteins. *Biochimica et Biophysica Acta (BBA) - Molecular Cell Research*.

[57] Yamamoto M., Clark J. D., Pastor J. V. (2005). Regulation of oxidative stress by the anti-aging hormone klotho. *Journal of Biological Chemistry*.

[58] Unger R. H. (2006). Klotho-induced insulin resistance: a blessing in disguise?. *Nature Medicine*.

[59] Emerling B. M., Weinberg F., Liu J. L., Mak T. W., Chandel N. S. (2008). PTEN regulates p300-dependent hypoxia-inducible factor 1 transcriptional activity through forkhead transcription factor 3a (FOXO3a). *Proceedings of the National Academy of Sciences of the United States of America*.

[60] Lim S. W., Jin L., Luo K. (2017). Klotho enhances FoxO3-mediated manganese superoxide dismutase expression by negatively regulating PI3K/AKT pathway during tacrolimus-induced oxidative stress. *Cell Death & Disease*.

[61] Kroemer G., Marino G., Levine B. (2010). Autophagy and the integrated stress response. *Molecular Cell*.

[62] Min S. Y., Ha D. S., Ha T. S. (2018). Puromycin aminonucleoside triggers apoptosis in podocytes by inducing endoplasmic reticulum stress. *Kidney Research and Clinical Practice*.

[63] Banerjee S., Zhao Y., Sarkar P. S., Rosenblatt K. P., Tilton R. G., Choudhary S. (2013). Klotho ameliorates chemically induced endoplasmic reticulum (ER) stress signaling. *Cellular Physiology and Biochemistry*.

[64] Chen T., Ren H., Thakur A. (2016). Decreased level of Klotho contributes to drug resistance in lung cancer cells: involving in Klotho-mediated cell autophagy. *DNA and Cell Biology*.

[65] Chen J., Zhang H., Hu J. (2017). Hydrogen-rich saline alleviates kidney fibrosis following AKI and retains Klotho expression. *Frontiers in Pharmacology*.

[66] Kuang X., Zhou H. J., Thorne A. H., Chen X. N., Li L. J., Du J. R. (2017). Neuroprotective effect of ligustilide through induction of *α*-secretase processing of both APP and Klotho in a mouse model of Alzheimer’s disease. *Frontiers in Aging Neuroscience*.

[67] Li L., Zhang M., Zhang L., Cheng Y., Tu X., Lu Z. (2017). Klotho regulates cigarette smoke-induced autophagy: implication in pathogenesis of COPD. *Lung*.

[68] Han S. W., Li C., Ahn K. O. (2008). Prolonged endoplasmic reticulum stress induces apoptotic cell death in an experimental model of chronic cyclosporine nephropathy. *American Journal of Nephrology*.

[69] Ahn K. O., Li C., Lim S. W. (2008). Infiltration of nestin-expressing cells in interstitial fibrosis in chronic cyclosporine nephropathy. *Transplantation*.

[70] Lim S. W., Jin L., Jin J., Yang C. W. (2016). Effect of exendin-4 on autophagy clearance in beta cell of rats with tacrolimus-induced diabetes mellitus. *Scientific Reports*.

[71] Lim S. W., Jin L., Luo K., Jin J., Yang C. W. (2017). Ginseng extract reduces tacrolimus-induced oxidative stress by modulating autophagy in pancreatic beta cells. *Laboratory Investigation*.

[72] Lim S. W., Hyoung B. J., Piao S. G., Doh K. C., Chung B. H., Yang C. W. (2012). Chronic cyclosporine nephropathy is characterized by excessive autophagosome formation and decreased autophagic clearance. *Transplantation Journal*.

[73] Lim S. W., Shin Y. J., Luo K. (2019). Effect of Klotho on autophagy clearance in tacrolimus-induced renal injury. *The FASEB Journal*.

[74] Hu M. C., Shi M., Gillings N. (2017). Recombinant *α*-Klotho may be prophylactic and therapeutic for acute to chronic kidney disease progression and uremic cardiomyopathy. *Kidney International*.

[75] Behera R., Kaur A., Webster M. R. (2017). Inhibition of age-related therapy resistance in melanoma by rosiglitazone-mediated induction of Klotho. *Clinical Cancer Research*.

[76] Chen T. H., Kuro O. M., Chen C. H. (2013). The secreted Klotho protein restores phosphate retention and suppresses accelerated aging in Klotho mutant mice. *European Journal of Pharmacology*.

[77] Leon J., Moreno A. J., Garay B. I. (2017). Peripheral elevation of a Klotho fragment enhances brain function and resilience in young, aging, and *α*-synuclein transgenic mice. *Cell Reports*.

[78] Shin Y. J., Luo K., Quan Y. (2019). Therapeutic challenge of minicircle vector encoding Klotho in animal model. *American Journal of Nephrology*.

